# Effect on implant drills and postoperative reactions for pre-extraction interradicular implant bed preparation during the COVID-19 pandemic and beyond

**DOI:** 10.1097/MD.0000000000029249

**Published:** 2022-08-19

**Authors:** Tian-Ge Deng, Ping Liu, Hong-Zhi Zhou, Yang Xue, Xue-Ni Zheng, Zhao-Hua Ji, Lei Wang, Kai-Jin Hu, Yu-Xiang Ding

**Affiliations:** a State Key Laboratory of Military Stomatology & National Clinical Research Center for Oral Diseases & Shaanxi Clinical Research Center for Oral Diseases, Department of Oral Surgery, School of Stomatology, The Fourth Military Medical University, Xi’an, China; b Department of Epidemiology, School of Public Health, Air Force Medical University, Xi’an, China; c Department of Female Mental Health, Xi’an Mental Health Center, Xi’an, China; d Department of Dental Implant, Xi’an Savaid Stomatolgy Hospital, University of Chinese Academy of Sciences, Xi’an, China.

**Keywords:** COVID-19, immediate implant, implant drill, postoperative reactions, surface roughness

## Abstract

The aim of the present study was to observe the abrasion of implant drills and postoperative reactions for the preparation of the interradicular immediate implant bed during the COVID-19 pandemic and beyond.

Thirty-two implant drills were included in four groups: blank, improved surgery, traditional surgery, and control. In the improved surgery group, a dental handpiece with a surgical bur was used to decoronate the first molar and create a hole in the middle of the retained root complex, followed by the pilot drilling protocol through the hole. The remaining root complex was separated using a surgical bur and then extracted. Subsequently, the implant bed was prepared. Implant drills were used in the traditional surgery group to complete the decoronation, hole creation, and implant-drilling processes. The tooth remained intact until the implant bed was prepared. The surface roughness of the pilot drill was observed and measured. Surgery time, postoperative reactions (swelling, pain, and trismus), and fear of coronavirus disease 2019 scale (FCV-19S) were measured and recorded, respectively.

Statistical analysis revealed significant difference with surface roughness among blank group (0.41 ± 0.05 μm), improved surgery group (0.37 ± 0.06 μm), traditional surgery group (0.16 ± 0.06 μm), and control group (0.26 ± 0.04 μm) (*P* < .001). Significant differences were revealed with surgery time between improved surgery group (5.63 ± 1.77 min) and traditional surgery group (33.63 ± 2.13 min) (*P* < .001). Swelling, pain, and trismus (improved group: *r* ≥ 0.864, *P* ≤ .006; traditional group: *r* ≥ 0.741, *P* ≤ .035) were positively correlated with the FCV-19S.

This study proved that a new pilot drill could only be used once in traditional surgery but could be used regularly in improved surgery. Improved surgery was more effective, efficient, and economical than the traditional surgery. The higher FCV-19S, the more severe swelling, pain, and trismus.

## 1. Introduction

The novel coronavirus disease 2019 (COVID-19) was first reported in Wuhan, China, in December 2019, which led to fear among individuals and impacted their oral health.^[1–4]^ Fear of COVID-19 was assessed using the fear of coronavirus disease 2019 scale (FCV-19S), which is a seven-item unidimensional scale with robust psychometric properties.^[5,6]^ All elective dental treatments were delayed, which increased the incidence of dental-related infections and emergencies. Some dental implant procedures are generally regarded as elective and non-emergency care.^[7]^ However, long-term delay in these procedures could lead to systemic and oral health problems. Other dental implant procedures were regarded as urgent procedures and dental emergency care, which might have an impact on aesthetics and function, which are usually associated with pain.^[7]^ These urgent conditions should be treated promptly. Therefore, patients who were diagnosed with a dental emergency, who could not delay extraction and required early restoration of aesthetics and function, should be considered for immediate implant placement.

Immediate implant placement can reduce the treatment duration, patient stress, and procedural costs.^[8,9]^ Moreover, the long-term survival and success rates of immediately placed implants are similar to those of implants placed using the conventional, delayed approach.^[10–12]^ However, precise positioning and angulation when performing initial drilling for immediate implant bed preparation are difficult at a multirooted molar site. Immediate implant placement in the multirooted molar area is associated with some challenges, such as large extraction sockets^[8]^ or a deficient height apical to the socket fundus.^[12]^ Furthermore, the interradicular bone septa may compromise the preparation of an ideal implant bed following tooth extraction, as drills easily deviate from their planned position and direction on the surface of the bone septa, resulting in unsatisfactory implant positioning.^[13]^

A pilot drill approximately 2.0 mm in diameter, usually represents the first osteotomy drill to be used for implant bed preparation.^[14,15]^ Precise drill positioning and angulation of the pilot drill play a key role in determining the precise positioning and angulation of sequential expanding drills for immediate implant bed preparation at the multirooted molar site, which determines the precise positioning and angulation of the dental implant. Currently, various technical methods, such as the use of surgical guide templates based on cone-beam computed tomography (CBCT) and computer-aided three-dimensional implant designs, facilitate precise implant bed preparation.^[16]^ However, the use of a surgical guide template does not completely prevent pilot drill deviation caused by the presence of bone septa. Some authors have described approaches for interradicular implant bed preparation at multirooted molar sites.^[17–19]^ These authors utilized implant drills^[17,18]^ or Linderman burs, and ultrasonic osteotomes^[19]^ for decoronation, implant bed preparation, and separation of the root complex, thereby ensuring precise positioning and angulation of the implant. However, despite the benefits of this approach, it is costly and time-consuming.

The aim of the present study was to observe improved surgery and traditional surgery in terms of surgery time, postoperative reactions, FCV-19S and degree of abrasion with pilot drills for immediate interradicular implant bed preparation at a multiroot molar site during the COVID-19 pandemic and beyond. This will further help to guide the clinical practice of immediate implants.

## 2. Materials and Methods

This observational study was used to observe the abrasion of pilot drills and postoperative reactions of improved surgery and traditional surgery for interradicular immediate implant bed preparation at a multirooted molar site at the Department of Oral Surgery, Stomatological Hospital of the Fourth Military Medical University from May 2020 to December 2020. This protocol was approved by the Institutional Review Board (IRB) of the Stomatological Hospital of the Fourth Military Medical University (IRB-REV-2020034). This study was conducted in accordance with the World Medical Association Declaration of Helsinki (version 2013). This study identifies the institutional and licensing committee that approved this study, including any relevant details, confirms that all studies were performed in accordance with relevant named guidelines and regulations, and confirms that informed consent was obtained from all participants. All relevant data will be verified to ensure that they are recorded accurately. All relevant data will be provided by corresponding authors.

### 2.1. Patient selection

All patients and their family members were required to COVID-19 related evaluations. Every patient wore a mask and had a review of the systems and the temperature checked before treatment. Patients who presented with any of the COVID-19 suspected positive symptoms mentioned should not be treated and given an appointment at least 2 weeks from the present date. Then, they underwent detection of COVID-19 viral nucleic acid assays before surgery.

Patients were included:

1. nonsmokers;
2. sufficient bone height and width apical to the socket fundus and interradicular bone septa for immediate implant placement without obvious inflammation;
3. the absence of risk factors, including osteoporosis, the use of bisphosphonates, acute hepatitis, myocardial infarction, diabetes, radiotherapy, chemotherapy, and immunosuppression treatment;
4. all patients had to provide negative COVID-19 viral nucleic acid assays 1 day before surgery;
5. all patients were followed up for at least 6 months.

Patients were excluded if they had active infections, severe periodontal disease, ankyloses, fused or converging roots, or unfavorable root position. The mandibular first molar was diagnosed as endodontically untreatable and recurrently acute or chronic pain, which could not delay extraction and required early restoration of mastication, and was screened for immediate interradicular implant bed preparation by the same experienced dentist.

The following parameters were recorded: age, sex, preoperative FCV-19S, tooth position, bone type, implant size, surgical method, pilot drill, surgical time, postoperative FCV-19S, postoperative reactions, and follow-up. Based on the mean FCV-19S of the pre-analysis with 12 patients (improved surgery group 13.30 ± 4.93 and traditional surgery group 19.67 ± 4.84), the shedding rate of the study sample was usually 20%, and the minimum sample size was 16 (PASS 11.0, Test for Two Means: Power = 0.9, α = 0.05). Sixteen patients (16 new pilot drills) were included in the improved and traditional surgery groups. Sixteen implants were placed immediately using the same implant system (Straumman, Basel, Switzerland) in the mandibular first molar region. CBCT (voltage: 220 V; power frequency: 50 Hz; input power: 660 VA; HiRes3D, Tsinghua Langshi Instrument Co. LTD, Beijing, China) and preoperative intraoral photographs of the 16 patients were also recorded before surgery.

### 2.2. Surgery procedure

In this study, antiseptic mouth rinses with 0.2% povidone-iodine and high-volume suction decreased the risk of disease transmission. The personal protective equipment must be used to ensure the safety of dentists and dental assistants. The dental high-speed, up-exhaust, air-driven handpiece with anti-retractive valves and surgical bur (ADZ-4, Northwest Medical Equipment, ShaanXi, China) parameters were rotation speed (300,000 rpm) and external irrigation (sterile water, 50 mL/min). Implant motor (NSK Surg Pro Motor System, Japan) parameters were contra-angle (20:1), rotation speed (1200 rpm), torque (45 Ncm), and external irrigation (Injection Saline, 40 mL/min). All the surgical procedures were performed by the same experienced surgeon.

### 2.3. Implant bed preparation

#### 2.3.1. Improved surgery group.

**Figure 1.**
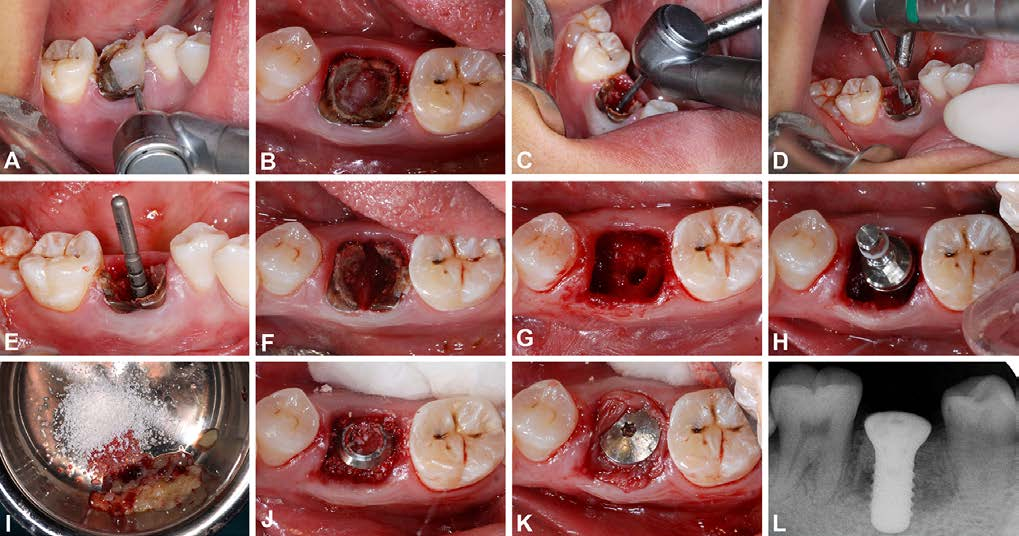
Pre-extraction interradicular implant bed preparation with improved surgery. (A) The first molar was decoronated at the level of the gingival margin using a dental high-speed, up-exhaust, air-driven handpiece with a surgical bur. (B) Occlusal view of the decoronated molar. (C) A hole (diameter: 2.5 mm) was created in the center of the retained root complex to the depth of the root furcation. (D) Drilling for the first implant bed preparation was performed through this 2.5-mm hole using a pilot drill (diameter: 2.2 mm). (E) The guiding rod revealed the precise positioning and angulation of the pilot hole. (F) Root complex was separated buccolingually. (G) Roots were extracted atraumatically without flap elevation. (H) A cylindrical screw-type dental implant (4.8 mm × 12 mm, wide neck, Straumman, Basel, Switzerland) was inserted. (I) Bio-Oss was mixed with CGF granules at a ratio of 1:1. (J) The gap between the socket ridge and implant was filled with the mixture of Bio-Oss and CGF. (K) The CGF membrane was fixed with a healing cap that covered the socket wound. (L) An apical image was taken immediately after surgery, revealing ideal implant positioning and appropriate filling of the gap with bone graft material. (CGF = concentrated growth factors.) (B, F, G, H, J, and K: mirror image.)

After the application of local anesthesia with articaine hydrochloride 2% and epinephrine tartrate 1:100,000 (Produits Dentaire Pierre Rolland, Merignac Cedex, France), the first molar was decoronated at the level of the gingival margin (Fig. 1A, B), and a hole (diameter: 2.5 mm) deep in the root furcation was created in the middle of the retained root complex using a dental high-speed, up-exhaust, air-driven handpiece with a surgical bur (Fig. 1C). The implant bed was initially prepared through this hole by using a pilot drill (diameter: 2.2 mm) (Fig. 1D). The retained root complex was used to guide the pilot drill, allowing precise drill positioning and angulation (Fig. 1E). Subsequently, the root complex was separated using a high-speed handpiece with a surgical bur (Fig. 1F), after which the roots were extracted atraumatically without flap elevation. The implant bed preparation protocols were performed in accordance with the manufacturer's instructions (Fig. 1G).

#### 2.3.2. Traditional surgery group.

A pilot drill was used to decoronate the first molar and perform initial drilling for immediate implant bed preparation penetrating through the middle of the retained root complex to the interradicular bone septa. After the drilling depth and angulation were confirmed, the implant bed preparation protocols were completed through the center of the retained root complex.^[17,18]^ Finally, the root complex was carefully extracted using a minimally traumatic elevator and forcep.

### 2.4. Implant insertion and socket grafting

Cylindrical screw-type dental implants were inserted in both groups (Fig. 1H). All implants were placed at a maximum torque of 35 Ncm. Immediately before the surgical procedure, concentrated growth factors (CGFs) were harvested from the patient's blood sample. The gaps between the socket ridge bone and implant were filled with the Bio-Oss and CGF mixture at a ratio of 1:1 (Fig. 1I, J). The sockets were covered with CGF membranes punched and fixed using healing caps connected to the implants (Fig. 1K). The apical films taken immediately after surgery revealed that the implants were in ideal positions, and the sockets were filled with bone grafts (Fig. 1L). Immediately after surgery, postoperative instructions were carefully explained to each patient. Each patient was prescribed oral antibiotics and analgesics for 2 days.

The surgery times for improved surgery and traditional surgery were recorded. The surgery time was defined as the time from decornating the crown to finishing the implant bed preparation.

### 2.5. The measurement of swelling, pain, and trismus

Postoperative reactions, such as swelling, pain, and trismus were measured and recorded by the same experienced surgeon. Swelling was the primary outcomes^[20]^ measured before surgery and at 1, 2, 3, 4, and 7 days post-surgery, including the distance from the mandibular angle to the external corner of the eye (distance MA-ECE), mandibular angle to the nasal border (distance MA-NB), mandibular angle to the labial commissure (distance MA-LC), and mandibular angle to the soft pogonion (distance MA-SP). Pain intensity was measured as the secondary outcome variable. The intensity of the primary pain variable was recorded using a 100 mm visual analog scale (VAS) from 0 (no pain) to 100 (worst pain imaginable). Each patient was invited to score their pain before surgery and at 1, 2, 3, 4, and 7 days after surgery.

Possible postoperative trismus^[21]^ was evaluated at maximum mouth opening (MMO) using a calibrated caliper (SL, 050805495, Limited Liability Company of Measurement Tool, Harbin, China) before surgery and at 1, 2, 3, 4, and 7 days post-surgery. The differences in the measurements compared with the baseline (before surgery) were assessed for each follow-up session.

### 2.6. Scanning electron microscopy (SEM)

**Figure 2.**
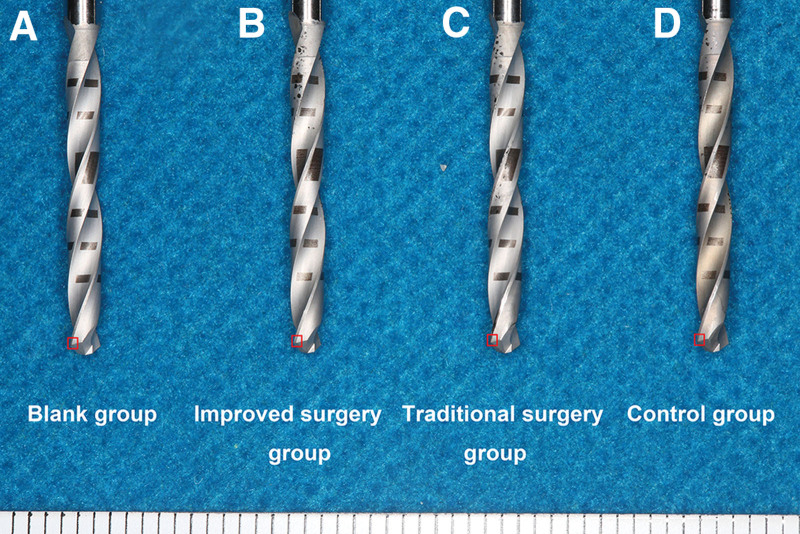
Gross observation of pilot drills. (A) Blank group. (B) Improved surgery group. (C) Traditional surgery group. (D) Control group. (Red box: cutting corners of pilot drills were defined as the regions of interest using scanning electron microscopy and atomic force microscopy.)

In this study, additional eight new pilot drills and eight discarded pilot drills were included, 32 pilot drills were submitted for evaluation by SEM (S-4800, Hitachi-High Technologies, Tokyo, Japan), which were included in four groups: blank group (eight new pilot drills with drilling bone 0 time), improved surgery group (eight pilot drills with drilling bone 1 time), traditional surgery group (eight pilot drills with drilling tooth and bone 1 time), and control group (eight discarded pilot drills with drilling bone 10 times) (Table 1). The cutting corner of the pilot drill was selected as the region of interest (ROI; 1 × 1 × 1 mm) (Fig. 2A–D). Photomicrographs were obtained for the cutting corners of pilot drills at ×50, ×500, ×1000, and ×5000 magnifications, to compare abrasion and loss of sharpness.

**Table 1:** Characteristics of 32 pilot drills among four groups.

	Blank group	Improved surgery group	Traditional surgery group	Control group
Pilot drills	Eight new pilot drills	Eight pilot drills with drilling bone 1 time	Eight pilot drills with drilling tooth and bone 1 time	Eight discarded pilot drills with drilling bone 10 times

### 2.7. Atomic force microscopy (AFM)

After SEM, all pilot drills were evaluated using AFM (Agilent 5500 SPM; Agilent Technologies, Santa Clara, CA). Three independent areas (50 × 50 μm) were measured at the cutting corners of the pilot drills. Images with 512 × 512 pixels were obtained in a constant force mode with PPP-NCL15 (Nano Sensor-S030104, ALT Technology Co., Ltd, Beijing, China) (spring constant of 0.2 N/m and tip radius of ≤10 nm) at a scan rate of 1.0 Hz. AFM micrographs were analyzed using the Pico Image Elements software (Agilent Technologies, Santa Clara, CA) to extract the surface parameters. The surface roughness of the pilot drills was quantified in the four groups in terms of the root mean square (RMS), which computed the standard deviation for the amplitudes of the surface.

### 2.8. Statistical analysis

All tests were performed using the SPSS software (version 17.0; SPSS, Inc., Chicago, IL). All graphs were generated using GraphPad Prism software version 6.0 for Windows (GraphPad Software, San Diego, CA). Quantitative variables, including surgery time, FCV-19S, RMS surface roughness, swelling, VAS pain score, and MMO, are expressed as mean ± standard deviation. Data normality for surgery time was tested using the Kolmogorov–Smirnov test. The homogeneity of variance for other data was tested using the homogeneity of variance test. Surgery time and FCV-19S were analyzed using an independent sample *t* test between the improved and traditional surgery groups. RMS surface roughness was analyzed using one-way ANOVA for the blank, improved surgery, traditional surgery, and control groups. Swelling, VAS pain score, and MMO were analyzed at many time points using repeated measures ANOVA. The correlations between FCV-19S and the changed distance MA-ECE, MA-NB, MA-LC, MA-SP, VAS pain score, and MMO were evaluated using Spearman correlation coefficient analysis. Multiple linear regression analysis was performed with changed distance MA-ECE, MA-NB, MA-LC, MA-SP, VAS pain score, and MMO as dependent variables and FCV-19S as independent variables. The significance level was set at *P* < .05 (two-tailed).

## 3. Results

A total of 16 patients (62.5% male, 37.5% female), with a mean age of 34.06 ± 5.32 years (range, 25–46 years), were included in this study; the average follow-up time was 10.25 ± 2.38 months (range, 6–14 months) (Table 2). All patients achieved clinical success during treatment. Submucosal emphysema or implant failure was not observed.

**Table 2:** Demographic and clinical characteristics of patients.

					Bone type	Implant size (SLA) (mm)							
Patients	Age (yr)	Gender	Preoperative FCV-19S	Tooth position		Diameter	Length	Type	Surgery method	Pilot drill	Surgery time (min)	Postoperative FCV-19S	Follow-up (mo)
No. 1	35	M	17	36	II	4.8	10	WN	Traditional surgery	Drilling tooth and bone 1 time	32	16	10
No. 2	46	F	18	46	III	4.1	12	RN	Traditional surgery	Drilling tooth and bone 1 time	35	23	12
No. 3	32	M	13	46	II	4.8	10	WN	Improved surgery	Drilling bone 1 time	8	5	14
No. 4	36	M	11	46	II	4.8	10	WN	Traditional surgery	Drilling tooth and bone 1 time	36	25	9
No. 5	34	F	12	36	II	4.8	10	WN	Improved surgery	Drilling bone 1 time	6	9	12
No. 6	30	F	11	46	II	4.8	12	WN	Improved surgery	Drilling bone 1 time	3	13	10
No. 7	28	M	14	46	II	4.8	10	WN	Improved surgery	Drilling bone 1 time	4	13	8
No. 8	37	M	15	46	II	4.8	12	WN	Traditional surgery	Drilling tooth and bone 1 time	30	27	7
No. 9	32	F	16	36	II	4.1	12	RN	Improved surgery	Drilling bone 1 time	6	15	11
No. 10	35	M	12	36	II	4.8	10	WN	Traditional surgery	Drilling tooth and bone 1 time	34	27	6
No. 11	42	F	14	36	III	4.1	12	RN	Traditional surgery	Drilling tooth and bone 1 time	36	25	10
No. 12	38	M	13	36	II	4.8	10	WN	Improved surgery	Drilling bone 1 time	5	15	8
No. 13	29	M	15	46	II	4.8	10	WN	Improved surgery	Drilling bone 1 time	8	17	11
No. 14	25	M	11	46	II	4.8	10	WN	Traditional surgery	Drilling tooth and bone 1 time	32	23	13
No. 15	30	F	16	36	II	4.1	10	RN	Traditional surgery	Drilling tooth and bone 1 time	34	35	14
No. 16	36	M	16	46	I	4.8	12	WN	Improved surgery	Drilling bone 1 time	5	18	9

FCV-19S = fear of coronavirus disease 2019 scale, RN = regular neck, SLA = sand blasted large-grit Acid-etched, surgery time = the surgery time was defined as the time from decornating the crown to finishing implant bed preparation, WN = wide neck.

### 3.1. Surgery time

**Figure 3.**
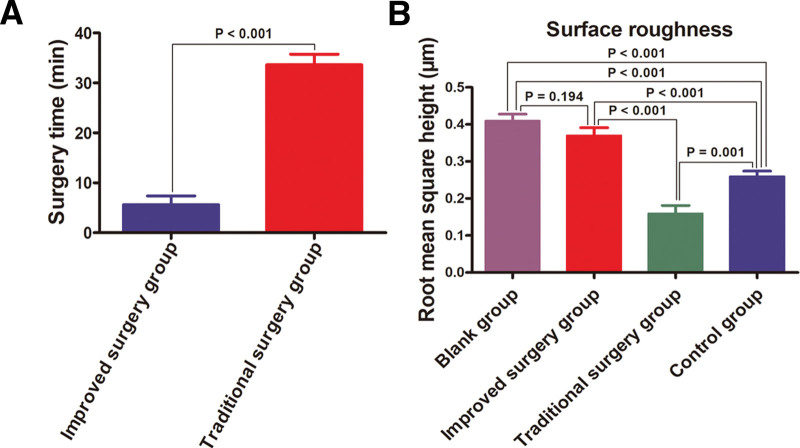
Comparison of surgery time and surface roughness among four groups. (A) Surgery time of improved surgery group versus traditional surgery group for pre-extraction interradicular implant bed preparation (*P* < .001). (B) Root mean square height (RMS) of surface roughness among the four groups using one-way ANOVA analysis (*P* < .001).

Surgery time of improved surgery group and traditional surgery group was 5.63 ± 1.77 min and 33.63 ± 2.13 min, respectively. Significant differences were observed in surgery time between the improved and traditional surgery groups (*P* < .001) (Fig. 3A).

### 3.2. The measured outcomes of swelling, pain, and trismus

**Figure 4.**
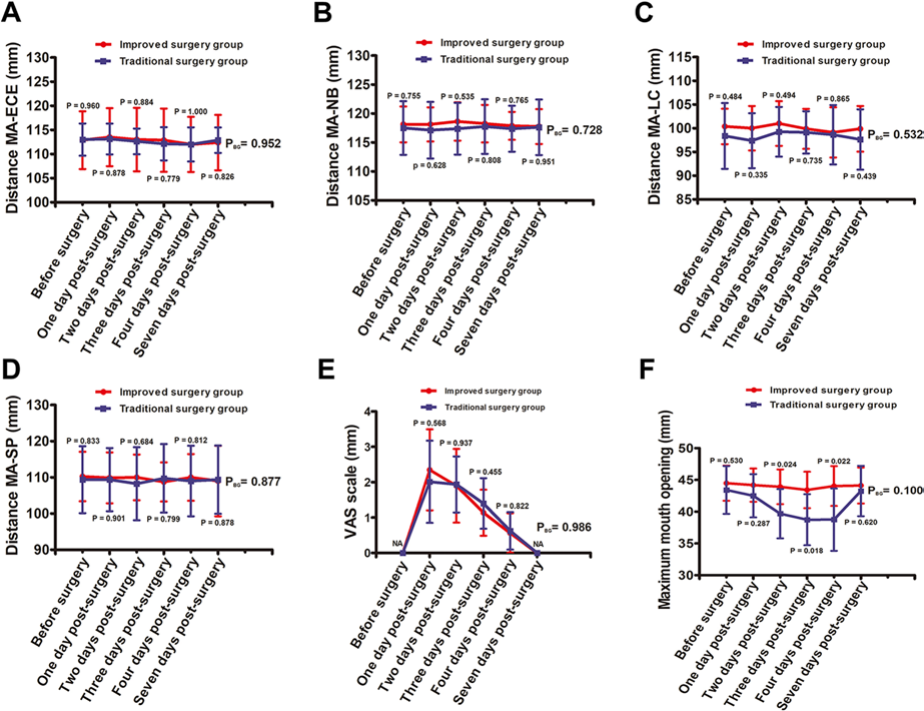
Comparison of the pre- and postoperative reaction between improved surgery group and traditional surgery group. (A–D) Comparison of the swelling values. (A) Comparison of distance MA-ECE (mandibular angle to external corner of the eye). (B) Comparison of distance MA-NB (mandibular angle to nasal border). (C) Comparison of distance MA-LC (mandibular angle to labial commissure). (D) Comparison of distance MA-SP (mandibular angle to soft pogonion). (E) Comparison of VAS (visual analog scale) pain score. (F) Comparison of maximum mouth opening. (The *P*_BG_ values: comparison between improved surgery group and traditional surgery group. Other *P* values: comparison between the groups at each time point. NA = not available.)

All patients completed the study without any serious postoperative reactions. Pre- and postoperative swelling were measured in the improved and traditional surgery groups (Fig. 4A–D). No significant difference was detected with distance MA-ECE (*P* = .952) (Fig. 4A), MA-NB (*P* = .728) (Fig. 4B), MA-LC (*P* = .532) (Fig. 4C), and distance MA-SP (*P* = .877) (Fig. 4D). No significant difference was detected in the VAS pain scores between the improved and traditional surgery groups (*P* = .986) (Fig. 4E). In addition, the MMO did not differ between the improved and traditional surgery groups (*P* = .100). However, significant differences were observed at 2 days (*P* = .024), 3 days (*P* = .018), and 4 days post-surgery (*P* = .022) (Fig. 4F).

### 3.3. The relationships between FCV-19S and postoperative reactions

**Figure 5.**
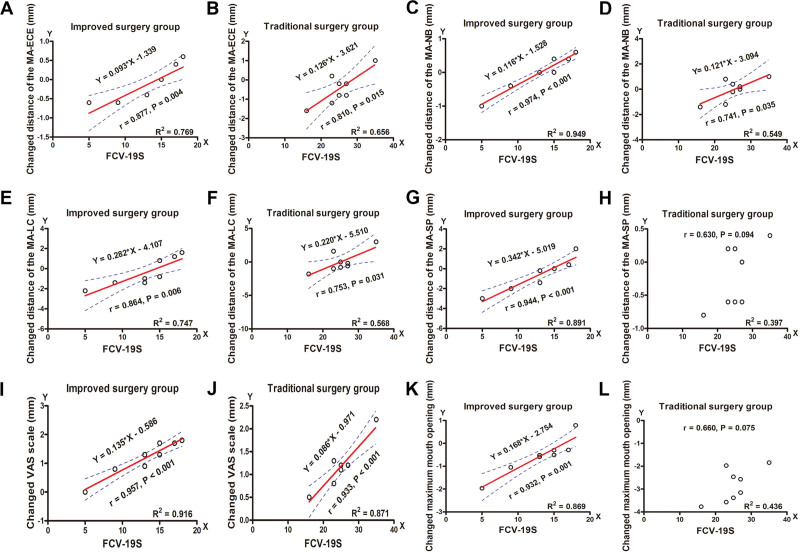
Relationships between FCV-19S and postoperative reactions. (A–G, I–K) FCV-19S were positively correlated with the changed distance MA-ECE, MA-NB, MA-LC, MA-SP, VAS pain score and maximum mouth opening in the improved and traditional surgery group, respectively. (H, L) No correlation was observed between FCV-19S and the changed distance MA-SP, maximum mouth opening in the traditional surgery group, respectively. (FCV-19S: fear of coronavirus disease 2019 scale).

All patients completed the pre- and postoperative FCV-19S. No significant difference was detected in preoperative FCV-19S between the improved surgery group (13.7 ± 1.83) and the traditional surgery group (14.25 ± 2.71) (*P* = .672). A significant difference was detected with postoperative FCV-19S in the improved surgery group (13.13 ± 4.29) and traditional surgery group (25.13 ± 5.30) (*P* < .001). Figure 5 shows that postoperative FCV-19S was positively correlated with the changed distance between MA-ECE (*r* = 0.877, *P* = .004; *r* = 0.810, *P* = .015), MA-NB (*r* = 0.974, *P* < .001; *r* = 0.741, *P* = .035), MA-LC (*r* = 0.864, *P* = .006; *r* = 0.753, *P* = .031), MA-SP (*r* = 0.944, *P* < .001), VAS pain score (*r* = 0.957, *P* < .001; *r* = 0.933, *P* < .001), and MMO (*r* = 0.932, *P* = .001) in the improved and traditional surgery groups (Fig. 5A–G, I–K). However, no correlation was observed between FCV-19S and the changed MA-SP distance (*r* = 0.630, *P* = .094) (Fig. 5H) and MMO (*r* = 0.660, *P* = .075) (Fig. 5L) in the traditional surgery group. The linear regression equations for these relationships are shown in Figure 5.

### 3.4. Scanning electron microscopy (SEM) analysis

**Figure 6.**
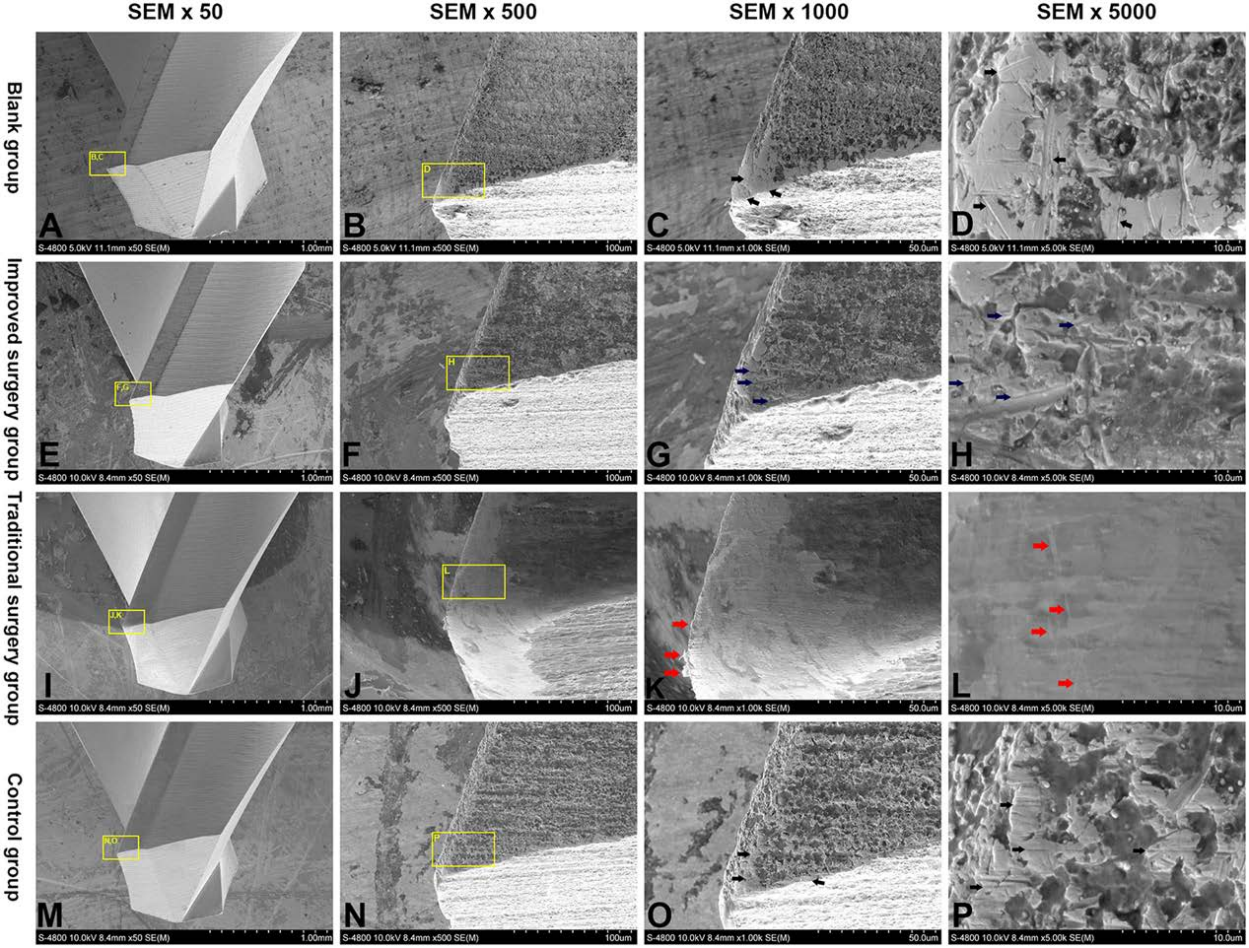
Scanning electron microscope (SEM) image of cutting corner of pilot drill. (A–D) Blank group. (A) Intact cutting corner was observed under low power SEM. (B–D) Irregular scratches of cutting corner was observed under high power SEM (black arrow). (E–H) Improved surgery group. (E) Almost intact cutting corner was observed under low power SEM. (F–H) A few parallel scratches of cutting corner was observed under high power SEM (blue arrow). (I–L) Traditional surgery group. (I) Loss of sharpness and abrasion area of cutting corner was observed, cutting corner became blunt and polished under low power SEM. (J–L) Metal subtraction and curling of cutting corner were observed under high power SEM (red arrow), and major irregular scratches were observed on polished abrasion area of cutting corner (red arrow). (M–P) Control group. (M) Almost intact cutting corner was observed under low power SEM. (N–P) Numerous parallel scratches of cutting corner was observed under high power SEM (black arrow).

In the blank group, a sharp major cutting corner of the new pilot drill was observed at 50 and 500 magnification (Fig. 6A, B), and a few irregular surface scratches (black arrow) of the new pilot drills were observed at 1000 and 5000 magnification (Fig. 6C, D).

In the improved surgery group, a sharp major cutting corner of the pilot drill was still observed at 50 and 500 magnification (Fig. 6E, F), and a few regular and parallel surface scratches (blue arrow) of the pilot drills were observed at 1000 and 5000 magnification (Fig. 6G, H).

In the traditional surgery group, abrasion and loss of sharpness were observed on the cutting corner of the pilot drill, and the sharp major cutting corner of the pilot drill disappeared at 50 and 500 magnification (Fig. 6I, J). Smooth and flat abrasion areas, plastic deformation edges, “Curling” metal laminas (red arrow), and blunting and small notched areas (red arrow) of pilot drill tips on the cutting corner were observed, and a few regular scratches on the smooth surface were also observed at 1000 and 5000 magnification (Fig. 6K, L).

In the control group, a sharp major cutting corner of the pilot drill was still observed, but not obviously, at 50 and 500 magnification (Fig. 6M, N), and many regular and parallel surface scratches (black arrow) of pilot drills on the cutting corner were observed at 1000 and 5000 magnification (Fig. 6O, P). Compared to surface abrasion among the four groups, the loss of sharpness and abrasion of pilot drills was progressive and increased from the blank group, improved surgery group, and control group to the traditional surgery group; the abrasion of the pilot drill was not evident in the improved surgery group, but was more evident in the traditional surgery group.

### 3.5. Atomic force microscopy (AFM) analysis

**Figure 7.**
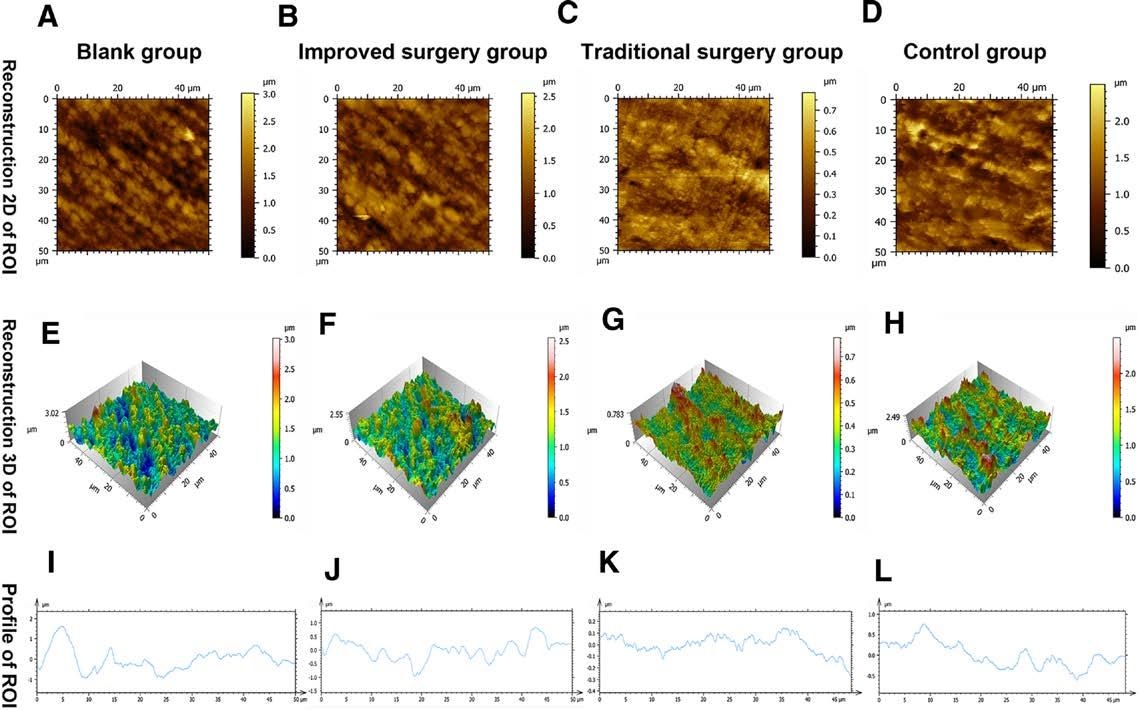
Atomic force microscope (AFM) surface morphology and roughness profile images of pilot drills with the region of interest (ROI). (A, E, I) Blank group. (B, F, J) Improved surgery group. (C, G, K) Traditional surgery group. (D, H, L) Control group. (A–D) Reconstruction of 2-dimension (2D) surface morphology. (E–H) Reconstruction of 3-dimension (3D) surface morphology. (I–L) Roughness profile images.

The 2D and 3D details of the ROI surfaces extracted from the four groups are shown, and the RMS surface roughness measured by AFM showed a varied display of peaks and valleys among the four groups (Fig. 7A–H). The roughness profiles of the ROI surfaces are shown for the four groups (Fig. 7I–L). RMS surface roughness were successively as followed: blank group (0.41 ± 0.05 μm), improved surgery group (0.37 ± 0.06 μm), traditional surgery group (0.16 ± 0.06 μm), and control group (0.26 ± 0.04 μm) (Fig. 3B).

One-way ANOVA revealed statistically significant differences in RMS surface roughness among the four groups (*P* < .001); there were statistically significant differences between the blank group and traditional surgery group (*P* < .001), blank group and control group (*P* < .001), improved surgery group and traditional surgery group (*P* < .001), improved surgery group and control group (*P* < .001), and traditional surgery group and control group (*P* = .001). However, no statistical differences were found between the improved surgery and blank groups (*P* = .194) (Fig. 3B). The RMS surface roughness was significantly higher in the improved surgery group than in the traditional surgery group. The RMS surface roughness of the improved surgery group appeared to be coarser surface with sharp-cornered and spiky morphologies, and the RMS surface roughness of the traditional surgery group appeared to be more uniform and flattened.

## 4. Discussion

The present purely observational study compared the loss of sharpness and abrasion of the pilot drill between two surgical methods of immediate implant bed preparation at the multirooted molar site: The first method received an improved surgical protocol in which a dental high-speed, up-exhaust, air-driven handpiece with a surgical bur was used to decoronate the first molar, create a hole deep to the root furcation in the middle of the retained root complex, and separate the root complex for tooth extraction. The implant bed was initially prepared through this hole using a new pilot drill, and another method received a traditional surgery protocol in which a new pilot drill was used to finish decoronation, hole creation, and pilot drilling. The assessment variables with the two surgical methods included surgery time, degree of abrasion of the pilot drill, and the measured outcomes of postoperative reaction and FCV-19S. The assessment variables showed that the improved surgery was more effective, efficient, and economical than traditional surgery. The higher FCV-19S, the more severe postoperative reaction.

Surgery time (duration) is an important variable for evaluating the surgical method.^[22–24]^ Shorter surgery time resulted in higher comfort, satisfaction, quicker recovery, excellent tolerability, and less postoperative reaction.^[25–29]^ The present study showed that the surgery time was significantly shorter in the improved surgery group than in the traditional surgery group. This result revealed that the improved surgery with a dental high-speed, up-exhaust, air-driven handpiece, and a surgical bur was more effective and efficient than traditional surgery with a surgical handpiece and a pilot drill driven by an electric motor at low speed. This showed that using a dental high-speed, up-exhaust, air-driven handpiece with anti-retractive valves and a surgical bur was a good choice to decoronate the first molar, create a hole deep to the root furcation in the middle of the retained root complex, and separate the root complex for tooth extraction, which could reduce complications such as submucosal emphysema and infection. Goyal et al^[30]^ reported that a rotary instrument driven by an electric motor for sectioning the tooth could reduce the surgery time (35 ± 11 min) more significantly than the piezosurgery technique (45 ± 16 min). Mozzati et al^[31]^ reported that a rotary instrument driven by an electric motor applied during odontotomy required a shorter surgery time (25 ± 5 min) than the piezosurgery technique (33 ± 5 min). The surgery time reported by Goyal et al and Mozzati et al with a rotary instrument was in line with the surgery time of the traditional surgery (33.63 ± 2.13 min). Different driving devices at different speeds resulted in different odontotomy efficiencies.^[15,19,32,33]^ In the traditional surgery protocol, the pilot drills are driven by an electric motor at a low speed for odontotomy. Therefore, the surgery time of the traditional surgery protocol was longer than that of the improved surgery protocol, which might result in greater discomfort, dissatisfaction, fear, and poor tolerability for the patient. However, the surgery time can be significantly reduced with less discomfort, pain, and swelling using an improved surgical protocol.

Postoperative reactions, including swelling, pain, and trismus, were used to evaluate the degree of surgical trauma.^[20,21,34,35]^ Some authors reported that measurements of postoperative reaction reflected the degree of surgical trauma after alveolar surgery^[24,27,34]^, which was similar to the present study. This study showed that the MMO was slightly lower in the traditional surgery group than in the improved surgery group at 2, 3, and 4 days post-surgery, and we deduced that the long surgery time of traditional surgery might result in jaw elevator muscle spasm and extended period of soft tissue retraction, which was in line with the study described by Menziletoglu et al.^[29]^ However, there were no significant differences in pain, swelling, and trismus between the improved and traditional surgery groups, which verified that the improved surgery did not increase surgical trauma. Some studies have reported that swelling and pain are related to bone damage, and reduced trauma indicates less postoperative reactions.^[24,27,28]^ The present study explained that the improved surgery using a dental high-speed, up-exhaust, air-driven handpiece with a surgical bur did not have bone damage and submucosal emphysema, and significantly improved the effectiveness of the surgery with less surgery time.

Moreover, no significant difference in postoperative reaction between the improved surgery protocol and the traditional surgery protocol might be due to the use of CGF to fill the peri-implant marginal gaps between the implant and socket walls and to cover the wound. Some studies have reported that CGF could not only accelerate soft-tissue healing and early bone formation but also reduce post-surgical edema, pain, trismus, and discomfort.^[36,37]^ Other literatures have shown that new bone formation was significantly increased in the defective bone region filled with CGF and Bio-Oss compared to Bio-Oss only.^[38,39]^

The FCV-19S is reliable and valid for assessing the fear of COVID-19 among individuals.^[5,6]^ The present study showed that FCV-19S was positively correlated with postoperative reactions in the improved and traditional surgery groups. We deduced that the COVID-19 pandemic affected patients’ physical and psychological health, caused physical damage to tissue and peripheral vessels from implant surgery, and increased localized inflammatory reactions associated with swelling, pain, and trismus. Psychological stress exert complex effects on the immune system, including immunosuppression, which could exacerbate postoperative inflammatory reactions and pain. The abovementioned explanations are in line with previous opinions.^[1–4]^ However, there were no correlations between FCV-19S and changed distance MA-SP and MMO in the traditional surgery group. We thought that the sample size might be relatively small and the power of the test was relatively low.

The cutting efficiency of a rotatory implant drill is clinically relevant, and the surface roughness and loss of sharpness at the cutting edge and corner of the drill can predict the cutting efficiency and durability.^[15,32,40–42]^ RMS deviation of the drill profile is an accurate way to evaluate the surface roughness, which depends on the average of a set of individual measurements of the peaks and valleys of a surface.^[14,43–45]^ In the present study, the RMS surface roughness, loss of sharpness, and abrasion of the pilot drills were evaluated using SEM and AFM. Our findings showed that comparisons of RMS surface roughness were as follows: blank group > improved surgery group > control group > traditional surgery group. The abrasion and loss of sharpness of the cutting corners of pilot drills were more notable in the traditional surgery group than in the control group. A new pilot drill with drilling bone 10 times must be discarded, according to the manufacturer's recommendations. However, a new pilot drill used only once in the traditional surgery group had to be discarded. However, no significant difference in abrasion and loss of sharpness of a new pilot drill was found between the blank group and the improved surgery group. The above analysis revealed that a new pilot drill could usually be used 10 times with the improved surgery protocol, which explained that the material wastage of improved surgery is one-tenth that of traditional surgery. Therefore, the improved surgical protocol using a pilot drill in combination with a dental high-speed, up-exhaust, air-driven handpiece, and surgical bur is more economical than the traditional surgery protocol. The protocols described in the literature^[33,46,47]^ were similar to the improved surgery protocol; however, their protocols did not quantify and compare the loss of sharpness and abrasion of implant drills.

Nevertheless, the limitations of this study are as follows: First, the tested samples of patients and implant drills were limited; second, the influence of disinfection and sterilization procedures were not considered for the measurement of surface roughness. Further studies must be conducted to evaluate these characteristics in larger samples of implant drills, correlated with the measurement of abrasion and heat generation during drilling procedures for pre-extraction interradicular implant bed preparation. Furthermore, the improved surgical protocol could not guarantee the accurate direction and angle of expanding drills because of the lack of a guide for the root complex; therefore, the improved surgical protocol required experienced surgeons to accomplish implant bed preparation.

## 5. Conclusion

Improved surgery with less discomfort, pain, and swelling was more effective, efficient, and economical than traditional surgery during the COVID-19 pandemic and beyond. The higher FCV-19S, the more severe swelling, pain, and trismus. Abrasion and loss of sharpness of the pilot drills were obvious in traditional surgery. A new pilot drill could only be used once in traditional surgery, but could be used regularly in improved surgery.

## Acknowledgments

All authors thank the Paperpal Preflight for English language editing.

## Author contributions

Conceptualization: Tian-Ge Deng, Kai-Jin Hu, and Yu-Xiang Ding.

Data curation: Tian-Ge Deng, Lei Wang, Ping Liu, Zhao-Hua Ji, and Hong-Zhi Zhou.

Formal analysis: Yang Xue, Xue-Ni Zheng, Zhao-Hua Ji, Lei Wang.

Investigation: Tian-Ge Deng, Lei Wang, Ping Liu, and Hong-Zhi Zhou.

Methodology: Tian-Ge Deng, Kai-Jin Hu, and Yu-Xiang Ding.

Resources: Kai-Jin Hu, Yu-Xiang Ding.

Supervision: Tian-Ge Deng, Kai-Jin Hu, Yu-Xiang Ding, and Lei Wang.

Validation: Tian-Ge Deng, Kai-Jin Hu, Yu-Xiang Ding, Lei Wang, and Ping Liu.

Writing – original draft: Tian-Ge Deng, Lei Wang, Ping Liu.

Writing – review and editing: Lei Wang, Kai-Jin Hu, Yu-Xiang Ding, and Hong-Zhi Zhou.
